# A Foreign Body Aspiration Leading to Pneumothorax: A Case of Airway Emergency

**DOI:** 10.7759/cureus.56489

**Published:** 2024-03-19

**Authors:** Ahmad Kamil Ahmad Fahmi, Ahmad Firdaus Habib Rahman, Anas Hadi Hishamuddin, Kanivannen Arasu, Avatar Singh Mohan Singh

**Affiliations:** 1 Otolaryngology - Head and Neck Surgery, Taiping Hospital, Taiping, MYS

**Keywords:** bronchus, otolaryngology emergency, pneumothorax, bronchoscopy, airway emergency, foreign body aspiration

## Abstract

Foreign body (FB) aspiration is a matter that should not be taken lightly. The presence of a foreign object might lead to hazardous complications, particularly in the pediatric population. These complications depend on the type and location of the aspirated object as the tracheobronchial tree has a very small diameter, and foreign bodies become stuck in the upper airways of children, causing stridor and sudden difficulty in breathing. Impaction of a foreign body in the right bronchial tree is more frequent due to the relatively straighter alignment of the right mainstem of the trachea, as opposed to the left side. Herein, we present a 10-year-old Malay boy who accidentally aspirated a pencil cap. An urgent computed tomography (CT) of the thorax revealed a suspicious intraluminal FB in the bronchus leading to pneumothorax and pneumomediastinum. He underwent a right bronchoscopy and a successful FB removal.

## Introduction

Foreign body (FB) aspiration is a common occurrence but can lead to devastating outcomes. Children aged one to three years formed the majority group with FB aspiration, leading to approximately 1000 deaths in children in the United States per year [1]. Clinical judgment in recognizing FB in the airways is paramount and must be recognized as an airway emergency. Recognizing the signs of the complications of FB aspiration must be a skill available to all medical practitioners. Imaging such as X-rays and CT scans must be utilized to help reach a diagnosis. Herein, we described a case of an FB aspiration leading to pneumothorax in the pediatric age group.

## Case presentation

A 10-year-old Malay boy with no known comorbidities presented with the accidental aspiration of the cap of a mechanical pencil. Following the incident, he experienced numerous instances of severe bouts of coughing. Later, he reported experiencing a sensation of a foreign object in his throat, along with intermittent difficulty breathing. Before reaching the hospital, he was well, active, tolerating orally, and did not experience any hoarseness or stridor.

Upon arrival at the emergency department, the child exhibited a positive and lively demeanor, with no signs of stridor or hoarseness. He was not tachypneic and spoke in full sentences. Upon lung auscultation, widespread rhonchi were detected on the left chest wall. A flexible nasopharyngolaryngoscopy revealed no obstructed upper airways, with both vocal cords exhibiting normal mobility and symmetry. There was no accumulation of saliva, and the subglottic region appeared clear. There was no foreign body (FB) seen.

**Figure 1:**
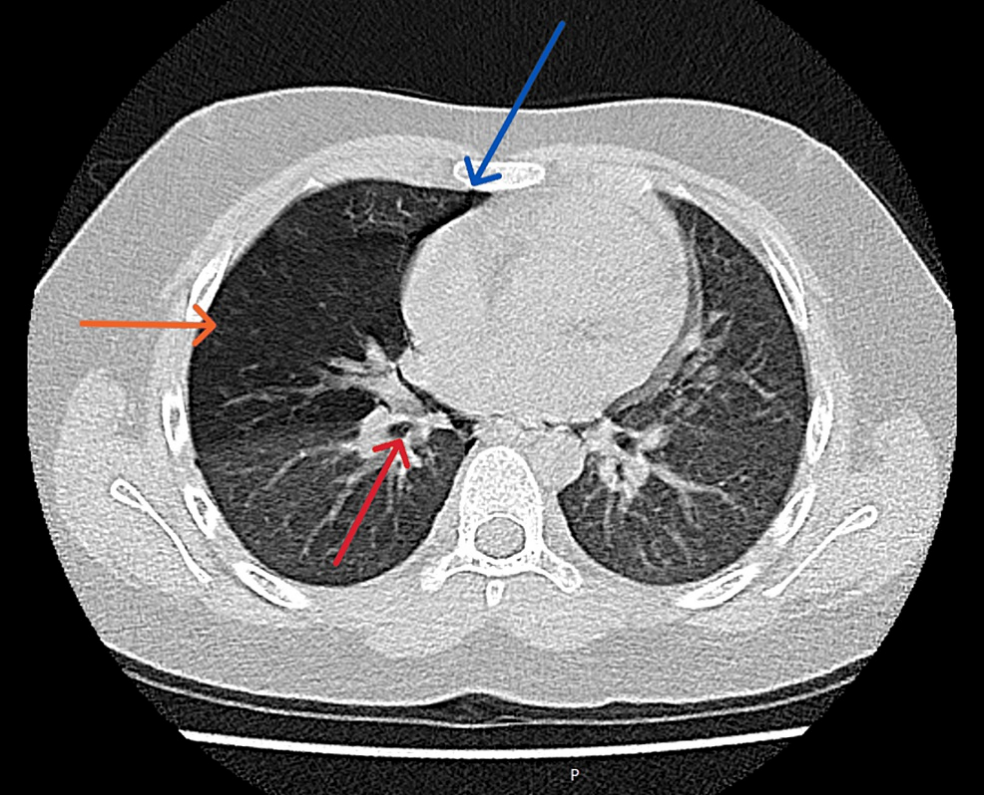
A slice of CT (axial view) showed the foreign body (red arrow), pneumothorax, pneumomediastinum (blue arrow), and middle lobe air trapping (orange arrow).

**Figure 2:**
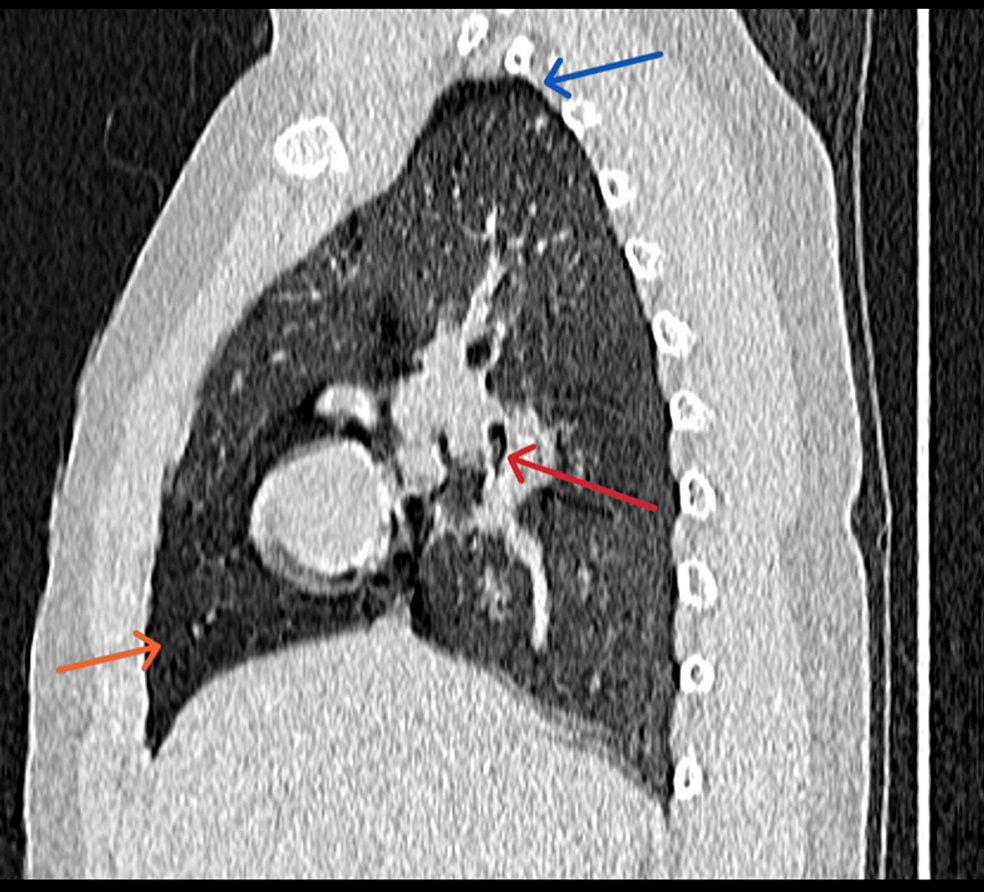
A slice of CT (sagittal view) showed the foreign body (red arrow), pneumothorax, pneumomediastinum (blue arrow), and middle lobe air trapping (orange arrow).

**Figure 3:**
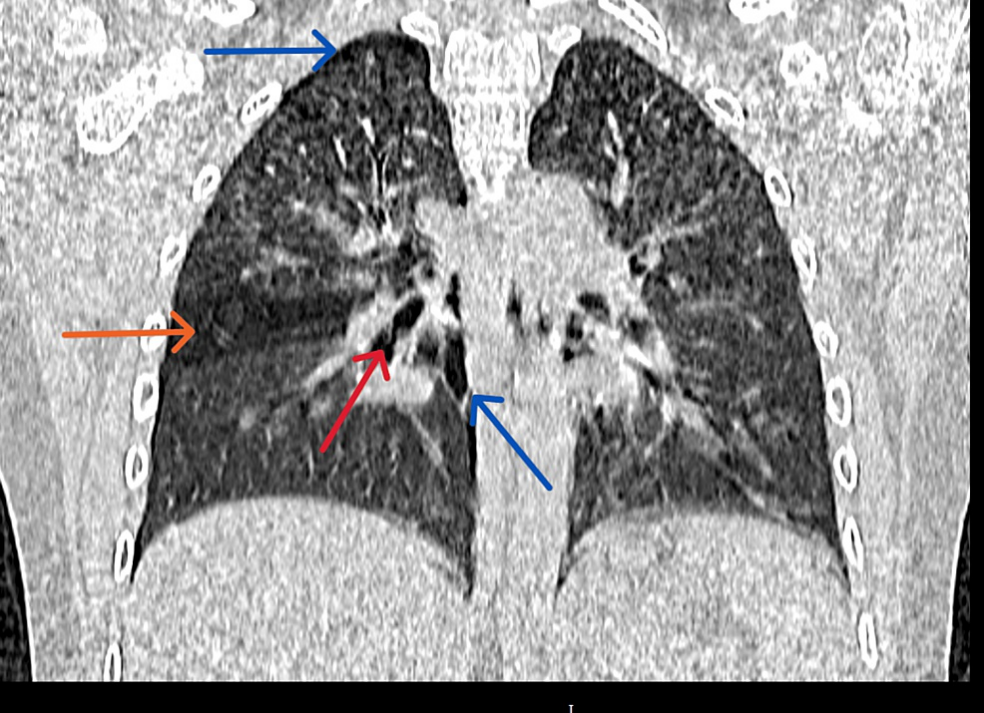
A slice of CT (coronal view) showed the foreign body (red arrow), pneumothorax, pneumomediastinum (blue arrow), and middle lobe air trapping (orange arrow).

The chest X-ray also revealed clear lung fields bilaterally. No discernible foreign object, pneumothorax, or pneumomediastinum were observed. An urgent CT Thorax was performed because of the notable history and physical examination findings. The CT thorax revealed abrupt tapering of the right middle lobe bronchus with a suspicious intraluminal FB in the bronchus intermedius causing right middle lobe air trapping. There was also right mild pneumothorax and pneumomediastinum (Figures 1-3).

**Figure 4:**
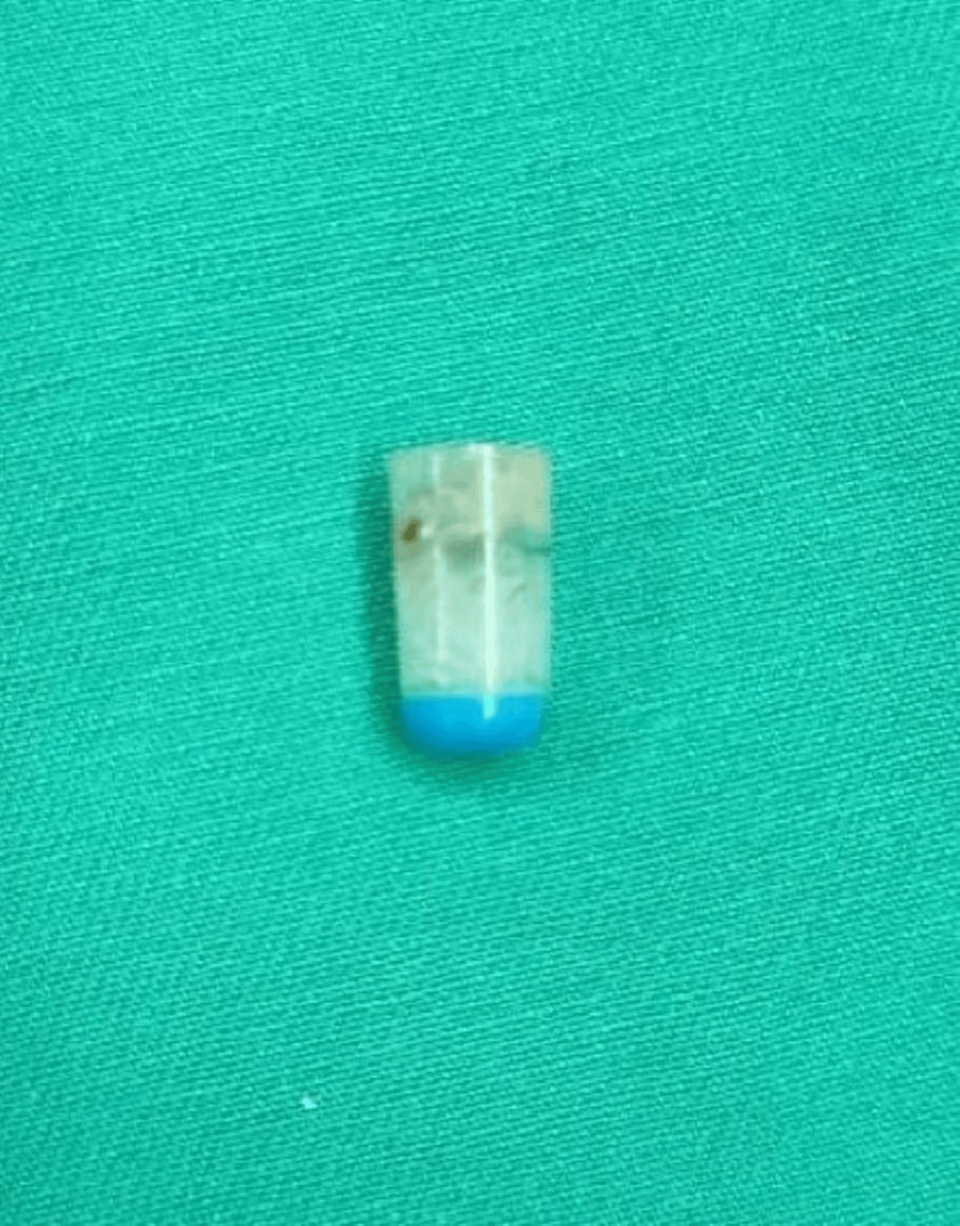
The intraluminal foreign body was removed and revealed to be the cap of a mechanical pencil.

He was then referred to a center with cardiothoracic expertise. He underwent a right bronchoscopy and foreign body removal. During the surgical procedure, the foreign object was located in the bronchus intermedius and extracted via bronchoscopy. The object was determined to be the cap of a mechanical pencil (Figure 4). A chest tube was not inserted.

Subsequently, he was closely monitored in the pediatric intensive care unit (PICU) for one day, followed by an additional three days of monitoring in the general ward. His overall state improved, and his pneumothorax was resolved. He was reviewed as an outpatient in the clinic and discharged in good health.

## Discussion

Aspiration of foreign body (FB) is the inhalation of FB into the larynx and tracheobronchial tree. It is more common in children but may occur in adults. Children aged one to three years form the majority group with FB aspiration [1]. It is uncommon for children of the age of the patient in the present case report to have such complications as they are more careful and alert in nature. However, the management in this age group is very similar to the overall management. Foreign body aspiration is a significant cause of morbidity and mortality in children. Restricted functional residual capacity and delicate cardiopulmonary status in children increase the likelihood of shunting and airway closing down. This will lead to poor ventilation and rapid hypoxemia [2]. Approximately 1000 children in the United States die each year due to the inhalation of foreign bodies [1].

The incidence of FB aspiration varies with dietary habits, with peanuts being the most common food-related FB besides seeds and food particles. Non-food-related FB were metal and plastic objects, such as toys, hair clips, pencil caps, ballpoint tips, and hair clips [3]. This report described a case of FB aspiration with a cap of a mechanical pencil. The child in this study is a 10-year-old boy, who uses this instrument of writing for daily activities and might accidentally aspirate the tip while biting on the mechanical pencil tip. Common aspirations in infants and toddlers are food-related FB, whereas non-food FB aspirations commonly occur in older children. The likelihood of infants and toddlers putting everything into their mouth and neural mechanism immaturity in swallowing and respiration contributes to the high frequency of inhaled FB in children. Boys were affected more than the girls [3,4]. This may be attributed to the personalities and nature of the children, but overall management between the two genders remained unaltered.

FB can get lodged in the airway from the supraglottis to the terminal bronchioles, which may vary in the symptoms and clinical problems at presentation. Around 80-90% of aspirated foreign bodies become lodged in the bronchi. Depending on the patient's age and physical position during aspiration, FB can get lodged in either bronchus. This can be explained by the angle of the trachea, and the two bronchi are similar until the age of 15 years. These angles change due to growth, so the right bronchus aligns more with the trachea. Aspirated FB that passes through the larynx will enter one of the bronchus and rarely cause life-threatening hypoxemia. However, life-threatening tracheal obstruction may occur if FB is too large to enter the bronchus [1].

Stridor, dyspnea, and cyanosis are not uncommon in laryngeal obstruction. In the case of a persistent cough, which may or may not be accompanied by an audible wheeze, FB aspiration must be suspected. A history of FB inhalation from patients, parents, and caregivers is not always available [5]. A thorough clinical examination is crucial in assessing patients with suspected aspirated FB for early diagnosis. Lung auscultations may reveal reduced breath sounds in the part of the bronchus where FB may be stuck [4]. These findings might be adequate to prompt urgent intervention to prevent further life-threatening complications.

Investigation by X-ray of the neck and chest is most important as it is more readily available in most healthcare facilities. Radiopaque FB can be quickly detected. Posteroanterior and lateral views must be included in the radiographic study. One of the most reliable findings in X-rays of recently aspirated patients with non-opaque bronchus is obstructive emphysema or air trapping. Features of atelectasis and pneumonic infiltrates may also be seen [4,6]. Based on several studies, the chest radiograph shows diagnostic accuracy ranging from 60% to 80% [6-8]. Other investigations to consider and may be helpful are computed tomography (CT) imaging and bronchoscopy.

FB confirmation and removal can be done by rigid or flexible bronchoscope. With proof of FB, removal can be done by bronchoscopy under general anesthesia. A flexible bronchoscopy may be used to attempt extraction. If it fails, then a rigid bronchoscope can be performed. General anesthesia with a relaxing agent and a ventilating bronchoscope are preferred. However, the risk of the FB getting dislodged further into the bronchial tree due to the jet ventilation must be considered [1,4]. If the bronchoscope fails to extract the FB, open techniques such as tracheostomy, bronchotomy, and thoracotomy may be considered. Open techniques of surgical methods might be performed in cases of distal bronchial placement, immobility, and impossible grasping of FB owing to the slippery surface or bronchial edema proximal to the FB [9].

Retained FB inside the bronchus can cause bronchial edema and bronchospasm. The drainage system distal to the impaction is impaired. Infection and atelectasis will be complicated further by bronchiectasis, bronchial stenosis, lung abscess, and bronchopleural fistula if no immediate action is taken. Subsequent loss of lung tissue function will impair growth and cause significant mortality and morbidity. Education and awareness are paramount in avoiding FB inhalation, especially for parents and caregivers, by restricting access to potentially dangerous small items with inhalational hazards toward children [3,4].

## Conclusions

FB aspiration is a potentially life-threatening occurrence. Hence, the authors of this study intend to highlight that the presence of a foreign object in the airway poses a significant and major airway emergency, particularly among children. An exhaustive history and thorough physical examination are crucial in determining the diagnosis and identifying any potential complications. If highly suspected history and abnormal findings are observed during the examination, it is advisable to have a low threshold in utilizing radiological means, such as X-rays and CT imaging, to reach diagnosis, particularly in the pediatric population. A delay in the diagnosis and prompt implementation of treatment may be fatal.
